# Supplementary material for: Effects of Dislocation Filtering Layers on Optical Properties of Third Telecom Window Emitting InAs/InGaAlAs Quantum Dots Grown on Silicon Substrates
Source: ACS Appl Mater Interfaces. 2024 Sep 13;16(38):51150–62. doi: 10.1021/acsami.4c12061 (PMC11440467; doi:10.1021/acsami.4c12061)
Supplement: Supplementary file 1 — am4c12061_si_001.pdf [file am4c12061_si_001.pdf]

Supporting Information to:

Effects of dislocation filtering layers on optical properties of third telecom window emitting InAs/InGaAlAs quantum dots grown on silicon substrates

Wojciech Rudno-Rudziński*[1], Michał Gawełczyk[2], Paweł Podemski[1], Ewelina Cybula[1], Sandeep Gorantla[3], Ramasubramanian Balasubramanian[4], Vitalii Sichkovskyi[4], Amnon J. Willinger[5], Gadi Eisenstein[5], Johann P. Reithmaier[4] and Grzegorz Sęk[1]

[1]*Department of Experimental Physics, Wrocław University of Science and Technology, St. Wyspiańskiego 27, 50-370 Wrocław, Poland*

[2]*Institute of Theoretical Physics, Wrocław University of Science and Technology, St. Wyspiańskiego 27, 50-370 Wrocław, Poland*

[3]*Łukasiewicz Research Network – PORT Polish Center for Technology Development, Stabłowicka 147, 54-066 Wrocław, Poland*

[4]*Technological Physics, Institute of Nanostructure Technologies and Analytics, CINSaT, University of Kassel, 34132 Kassel, Germany*

[5]*Electrical and Computer Engineering Department and Russell Berrie Nanotechnology Institute, Technion-Israel Institute of Technology, Haifa 32000, Israel*

While growing and characterizing unburied QDs is a common practice, it is well known[1-3] that the QD morphology undergoes a significant modification after covering with the barrier material due to diffusion processes, which leads to final QD geometry far from that of unburied ones. Additionally, QD sizes estimated by atomic force microscopy are typically overestimated due to convoluting the sample and tip geometries. For that reason, we treat transmission electron microscopy data as a more reliable source of input parameters for calculations, despite the drawbacks, such as small population of probed dots or uncertainty of cross-section position. Still, AFM images on unburied dots should reflect some characteristics of buried ones, especially when they are compared between the samples.

We have performed the AFM measurements using a Nanosurf Flex AFM with a Nanosurf Stat0.2LauD cantilever and Nanosurf C3000 control software. Surface topography was conducted in static force mode (20 nN) at room temperature in air, over a measurement area of 0.5 µm × 0.5 µm with a resolution of 256 points. Figure S1 presents the image for the reference sample, showing very high density of rather uniform and slightly elongated QDs, as expected for the InAs/InP dots optimized for laser applications.

* Corresponding author email: wojciech.rudno-rudzinski@pwr.edu.pl

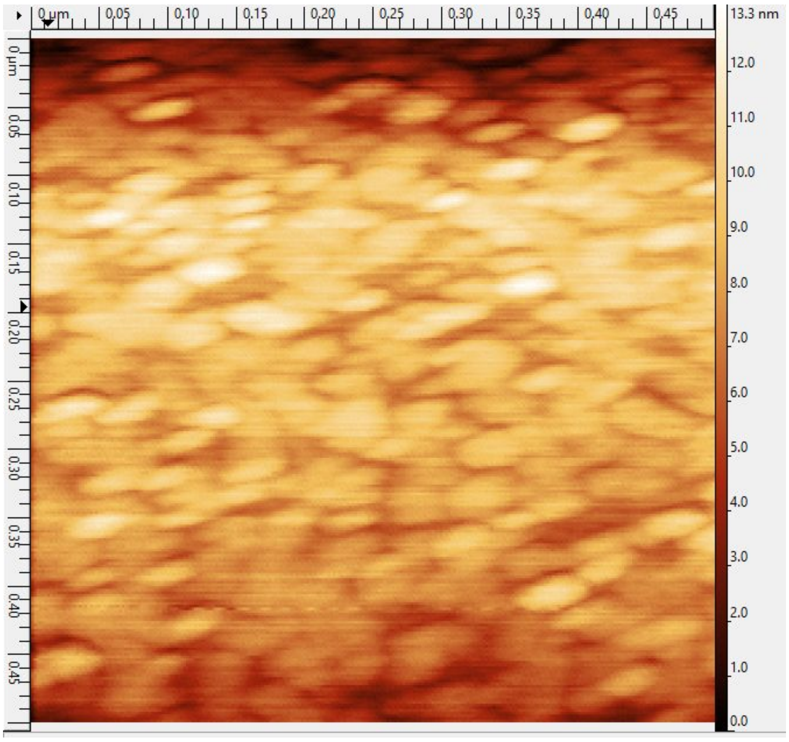

Fig. S1. AFM image for the reference sample.

Obtaining clear images for Si-based samples was much more difficult, due to at least two factors: very high spatial inhomogeneity along the surface and a complicated structure of atomic steps of various heights. One of the better images for the Sample B is shown in Fig. S2. Besides the population of dots similar to those in the reference samples (although with slightly lower surface density), there are also larger amalgamation of dots, probably responsible for the optical response of the second population also in the buried dots.

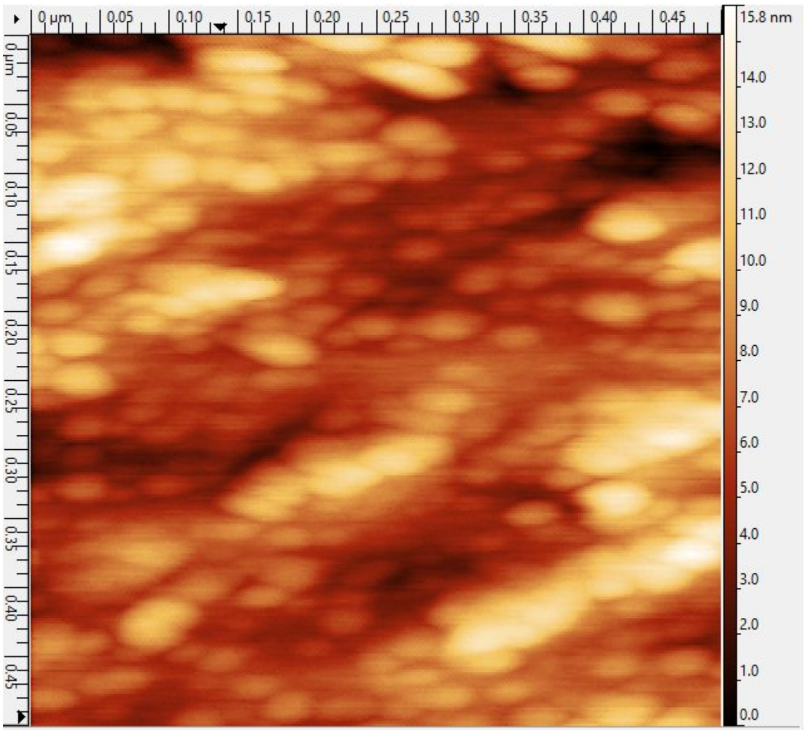

Fig. S2. AFM image for the sample B.

1. Appl. Phys. Lett. **81**, 1195 (2002); https://doi.org/10.1063/1.1500778
2. J. Cryst. Growth **251,** 155 (2003); https://doi.org/10.1016/S0022-0248(02)02407-7
3. J. Nanoparticle Res. **6**, 407 (2004); https://doi.org/10.1007/s11051-004-4704-6
